# Ribose Intake as Food Integrator: Is It a Really Convenient Practice?

**DOI:** 10.3390/biom12121775

**Published:** 2022-11-29

**Authors:** Roberta Moschini, Francesco Balestri, Mario Cappiello, Giovanni Signore, Umberto Mura, Antonella Del-Corso

**Affiliations:** 1 Biochemistry Unit, Department of Biology, University of Pisa, Via San Zeno, 51, 56127 Pisa, Italy; 2 Interdepartmental Research Center Nutrafood “Nutraceuticals and Food for Health”, University of Pisa, 56124 Pisa, Italy

**Keywords:** ribose, ribose-5-phosphate, ribose intake, protein glycation

## Abstract

Reports concerning the beneficial effects of D-ribose administration in cardiovascular and muscle stressful conditions has led to suggestions for the use of ribose as an energizing food supplement for healthy people. However, this practice still presents too many critical issues, suggesting that caution is needed. In fact, there are many possible negative effects of this sugar that we believe are underestimated, if not neglected, by the literature supporting the presentation of the product to the market. Here, the risks deriving from the use of free ribose as ATP source, forcing ribose-5-phosphate to enter into the pentose phosphate pathway, is emphasized. On the basis of the remarkable glycation capacity of ribose, the easily predictable cytotoxic effect of the molecule is also highlighted.

## 1. Introduction

Nucleotides, besides being relevant precursor in the nucleic acid (NA) synthesis, represent the most advanced goal of metabolic evolution in terms of ready to use chemical energy storage; they contain two phosphoanhydridic bonds, whose cleavage, under proper catalytic action, may provide the driving force of endergonic reactions needed for cell life. We can mention, just as an example, the UTP role in the generation of UDP-glucose, the specific precursor for the glucose storage as glycogen or the extraordinary concerted function of GTP as energy donor in the ribosomal machinery for protein synthesis and, lastly, the ubiquitous ATP involvement as energy supplier in innumerable and so different energy requiring processes. Indeed, ATP is synonymous of cell energy and the phosphorylation status of the adenylic nucleotides (i.e., AMP, ADP, and ATP) is taken as an index (i.e., the energy charge) of the energetic status of the cell. Thus, the attention of researchers (as well as entrepreneurs) in finding new strategies to favor the replenishment of the nucleotides in general and, in particular, of adenylyl nucleotides (both in terms of their levels and of their highest phosphorylation status) is understandable. We are so used to seeing the ribose scaffold in relevant biological molecules as nucleotides and nucleic acids that not thinking that D-ribose enters as a precursor of their synthesis will sound almost inconceivable. However, it seems that such an apparent “ready to use” precursor for nucleotide synthesis is mainly disregarded in the metabolic implant, being glucose, through its phosphorylation at C6, the preferred precursor of the ribofuranosidic scaffold of nucleotides [1]. Nevertheless, as underlined below, D-ribose intake has been proposed as a strategic approach to increase levels of ATP. If we simply consider the widespread “commercial propaganda” about the beneficial effects of D-ribose in the human metabolism, we may be convinced that this metabolite is essential for an energy-filled, healthy, and enjoyable life. Emphasis on this aspect appears in many commercial advertisements, highlighting the fact that ribose, being part of nucleotides, has the potential to enhance ATP availability. Thus, ribose intake is suggested to energize healthy subjects undergoing physical exercise. However, with a basic insight into metabolic processes, it is not difficult to challenge the commercial presentation of this “fantastic” food integrator, even if allegedly supported by literature references [2,3,4,5,6]. The main concern arising from many of these presentations is related to the ambiguity on the goal of the proposed treatment and the inadequacy in presenting what we can define as the “unfriendly features” of ribose. In fact, it is rather ambiguous whether ribose intake should improve the energy charge or the nucleotide pool level or both. On the other hand, the possible adverse effects linked to the reactivity of the ribose molecule are essentially neglected. Thus, we want to point out that, as presented, the energy injection that should occur in healthy active people (children, runners, body builders, sportsmen), deriving from D-ribose, doesn’t have, yet, any strong scientific base allowing the proposal and promotion for indiscriminate consumption of the sugar. Conversely, the risk of damage arising from circulating ribose is firmly standing. As potential customers rather than as scientists, we were surprisingly worried in learning of the actions undertaken by ribose Producers at the level of European Commission to advocate for the integration of this molecule in the formulations of a variety of edible products, from energy bars and drinks, to biscuits, flavored drinks, fruit juice, vegetable juice, and yogurt [7]. We do not know whether such actions are still proceeding, but certainly the topic is worth of attention by EU governments. Thus, in this review, some considerations are made on what is loudly claimed being advantageous, and what may be subtly dangerous regarding a ribose dietary intake by healthy people.

## 2. Ribose Metabolism and Nucleotides Synthesis

The preferential choice of the ribose scaffold as a building block for biomolecules is self-evident simply by observing its recurring presence in molecules, such as nucleotides and nucleic acids, which are so relevant for cell life. The reliability of molecules is considered as a factor possibly influencing their recruitment as privileged precursors of metabolic pathways. This is the indubitable case of the pyranosidic structure of glucose, whose reliability [8] but also its minimal, even though possible, glycating activity [9], led glucose to be chosen as the privileged precursor of bioenergetics pathways. The opposite is the case, following the same reasoning approach, for the use of ribose as metabolic precursor for nucleotide synthesis. Due to the structural unreliability of ribose and due to its the remarkable glycation ability, this sugar should have appeared ab initio too risky in supporting a relevant goal as the nucleotide synthesis. Then, the pentose phosphate pathway (PPP), jointly to its function of replenishment of reducing equivalents (i.e., NADPH formation), is widely recognized as the mainstream of ribose-5-phosphate (R5P) synthesis [10], through the use of glucose-6 phosphate as precursor. This unavoidable precursor of de novo nucleotide synthesis is an intermediate of the reversible reaction network of PPP. These reactions modulate the overall PPP by recycling the C3 to C6 glucose-derived carbon atoms into the main glycolytic/gluconeogenic flux. Thus, due to their reversibility, they represent the access door of glycolysis intermediates (i.e., glyceraldehyde-3-phosphate and fructose-6-phosphate) to R5P generation [11].

R5P would also directly enter PPP by the ATP dependent phosphorylation of D-ribose catalyzed by ribokinase (RK). This enzyme, which belongs to the PfkB family of carbohydrate kinases [12] has been characterized in human [13,14] and displays most of the features of the *E. coli* RK in terms of phosphate and metal ion activation. Nevertheless, the human enzyme exhibits a reduced efficiency with respect to bacterial RK, with K_M_ values for D-ribose of at least one order of magnitude higher (approximately 2 mM versus 0.2 mM) [15,16]. Furthermore, a substrate inhibition by ribose is observed, which may limit R5P formation [13]. Much more evident is the lack of efficiency of RK when compared to adenosine kinase (AdoK), another member of the PfkB carbohydrate kinase family. This enzyme shares with RK structural elements, regulatory properties, and the ability to recognize as substrate the C5-hydroxyl group of the ribofuranoside scaffold (here linked to the purine base). The K_M_ value of the human AdoK for adenosine is in the range of µM or sub-µM levels (0.1 to 3 µM), from two to three order of magnitude lower than of RK for D-ribose [17,18,19]. Besides possible peculiar differences between these strongly related family members, the poor effectiveness of RK may certainly be ascribed to the unreliability of the D-ribose, mostly present in solution in its pyranosidic form (80%) [20,21], which is less adequate as substrate for RK action [1]. In any case, R5P may eventually be formed upon RK action. This is the route invoked by ribose “supporters” to rationalize the beneficial features of exogenously supplemented ribose entering the glycolytic/gluconeogenic pathway and, most importantly, the nucleotides synthesis. In this regard it is worth noting that the need of this ATP-dependent activation step for ribose to enter metabolic routes excludes any possible advantage of ribose with respect to glucose as ergogenic nutrient. On the other hand, with some exceptions, ribose alone is unable to sustain cell growth [22,23,24]. Therefore, ribose would exclusively assume the possible role of precursor of nucleotides synthesis.

Now, the relevant questions are why and when this metabolic opportunity should be forced and, most importantly, its price.

## 3. The Claim of Ribose Intake as an Ergogenic Practice

The ergogenic effect of ribose comes out from studies in which the ability of the cell to warrant the homeostasis of ATP is somehow compromised. This is due to either a pathological status or an excessive energy expenditure. Evidence has been reported from studies on animals and humans for which ribose treatment may be helpful in cardiovascular diseases. An increase in the rate of ATP recovery in myocardium after ribose intravenous injection was observed in rats undergoing oxygen reperfusion after myocardial ischemia [25]. A similar effect was reported in a canine model for myocardial ischemia, in which the level of ATP, accounting for 50% of the pre-ischemic level, was restored after 24 h to 85% in animals subjected to ribose infusion [26]. Moreover, human subjects suffering of cardiopathic dysfunctions such as ischemia, cardiomyopathy, and hypertrophy, were beneficially affected by ribose administration [27,28,29].

D-ribose intake was successfully used to ameliorate symptoms in the case of fibromyalgia and chronic fatigue syndrome [2,30]. In a case report of a patient affected by myoadenilate deaminase deficiency, a recovery of ATP depletion was observed after ribose treatment [31]. This observation was, however, not supported by other case reports referring to the same pathological status [32].

With such evidence, the rational base of the reports claiming the beneficial effect of an external supplement of ribose generally referred to a failure of the oxidative branch of PPP, unable anymore to replenish R5P levels adequate for the de novo synthesis and “salvage” of nucleotides. This problem is very stimulating and worth being experimentally furthered to give answers to the above questions concerning why and when ribose intake may be beneficial.

The benefits that ribose may offer in a number of pathological situations has been exhaustively reviewed [33]. However, no research has been carried out regarding some problems that the ribose treatment may, at least theoretically, promote. The word “glycation”, highly expected to occur talking on ribose, does not appear all over the text. Much more attention was finally posed on this aspect in a very recent review on this topic [34].

Besides some attempts tailored to verify the potential usefulness of ribose intake to ameliorate the distress in pathological conditions, ribose treatment was widely tested to prove the advantages it could have for healthy people to ameliorate their physical performance. There is evidence that the rate enhancement, if any, of ATP resynthesis occurring after repeated physical exercise does not imply any improvement in the performance score [35,36,37,38]. However, a number of reports on healthy subjects underline the advantage of taking ribose [39,40,41,42]. It is in this literature frame that the idea to administer ribose to healthy people as energizer emerged, giving the scientific support for commercial spots to include ribose as a dietary integrator. While we will return later on this argument, we believe useful at this point, since the ribose intake is connected to the PPP, to have an insight on this pathway.

## 4. Getting Closer to PPP

The relevance of PPP as a source of reducing power (i.e., NADPH) and nucleotides precursor (i.e., R5P) is an assessed point for biochemistry students. However, how critical is this pathway for the proper function of cellular and organism health is probably underestimated [43].

### 4.1. The Oxidative Branch of PPP

**Figure 1:**
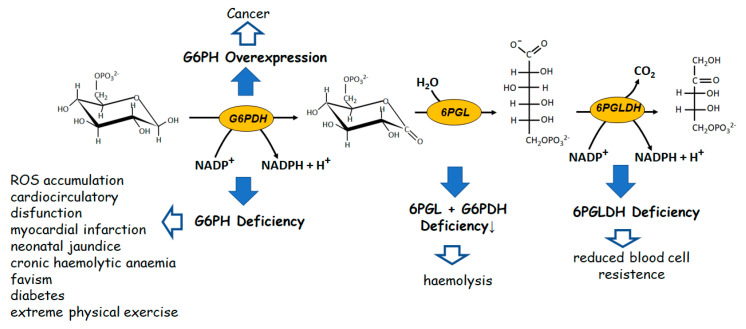
The oxidative branch of PPP. Alterations in enzymatic activities are connected and/or ob–served in the indicated pathological and stressful conditions, as mentioned in the text.

Looking at the oxidative irreversible part of PPP, apparently devoted to NADPH replenishment (Figure 1), the failure of glucose-6-phosphate dehydrogenase (G6PDH) activity, the enzyme which catalyzes the first and limiting step of the pathway, may determine dramatic pathological situations [44]. The deficiency of this enzyme derives from a genetic disorder, especially pronounced in specific world regions, characterized by several point mutations with a wide phenotypic heterogeneity [45,46].

Such a deficiency leads to a number of severe pathophysiologic states, such as neonatal jaundice [47], chronic non-spherocytic haemolytic anaemia [48], predisposition to haemolytic crisis induced by drugs or infections [49,50,51]. The pathology defined as “favism”, in which compounds present in fava beans, such as divicine and isouramil are inducers of haemolysis, is emblematic [52]. The haemolysis linked to G6PDH deficiency may also be triggered in diabetes, in myocardial infarction, or even during extreme physical exercise [53,54,55]. Cardio circulatory dysfunctions also have been related to deficiency of G6PDH activity. Also in this case, the effect was ascribed to a decrease of NADPH levels, which leads to the loss of modulation of the redox status of endothelial cells, which in turn induces ROS accumulation [56,57,58]. Alterations at level of 6-phosphogluco-δ lactone dehydrogenase (6PGLDH), which catalyses the second irreversible step of PPP, are rarer. Deficiency of this enzyme have been reported to be associated to a reduction of red blood cell resistance [59,60]. However, possibly since it is not a limiting step for G6P flux into PPP, the loss of approximately 50% to 80% in 6PGLDH activity does not determine severe consequences as it occurs for the G6PDH deficiency.

When 6-phosphogluco-δ lactone lactonase (6PGL) deficiency occurs on top of G6PDH deficiency, the situation may become more critical. In fact, in polymorphic variants of G6PDH in which no chronic hemolysis takes place, the concomitant lack of 6PGL may induce hemolysis [61]. Also, overexpression of G6PDH is associated with pathological states. This is the case, for instance, of the metabolic support offered by the over activity of the enzyme to cancer cell growth [62,63].

### 4.2. The Non-Oxidative, Reversible Branch of PPP

**Figure 2:**
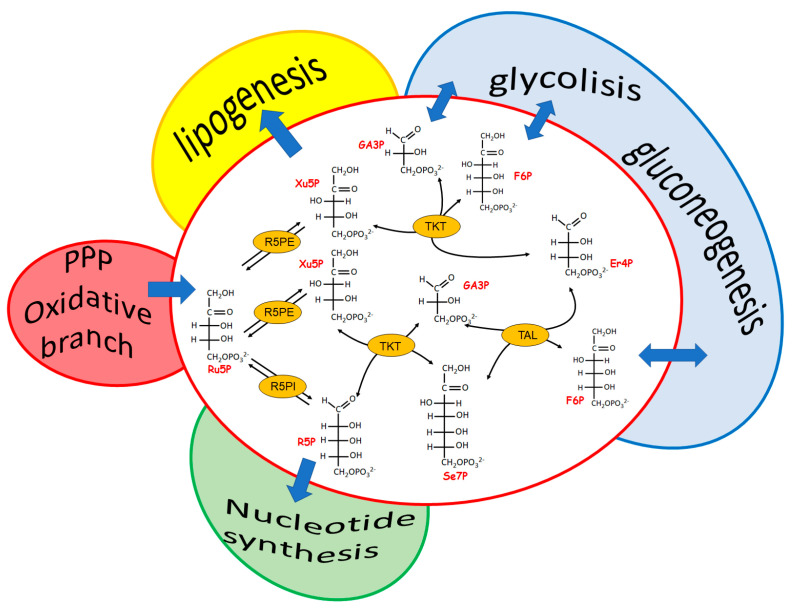
The reversible branch of PPP. The links among the reversible branch of PPP and relevant metabolic pathways are highlighted. Ru5PI, Ru5P-isomerase; Ru5PE, Ru5P-epimerase; TKT, trans–ketolase; TAL, transaldolase.

The most evident role of the reversible branch of PPP is to modulate recovery of pentose sugars to feed, through glucose-6P resynthesis, the oxidative PPP branch when reducing power, rather than R5P-linked nucleotide synthesis, is required. This pathway consists of four enzymes: two isomerases and two transferases. The isomerases refer to a ribulose-5-phosphate isomerase (Ru5PI), converting ribulose-5P (Ru5P) to R5P and a Ru5P epimerase (Ru5PE) converting Ru5P to xylulose-5P (Xu5P). The transferases refer to a transketolase (TKT) and a transaldolase (TAL). TKT is a thiaminepyrophosphate-dependent enzyme able to transfer a two-carbon group (a glycolaldehyde residue) from an α-ketosugar to an aldose, as is the case of the reactions between Xu5P and R5P, generating glyceraldehyde-3P (Ga3P) and sedoeptulose-7P (Se7P), and between Xu5P and erythrose-4P (Er4P), generating Ga3P and F6P. Finally, TAL catalyzes the transfer of a three carbon group (dihydroxyacetone residue) from an α-ketosugar (i.e., F6P and Se7P) to an aldose (i.e., Ga3P and Er4P) (Figure 2).

All of the reactions of this branch of PPP are reversible. However, nothing is more wrong than thinking of this pathway as a simple sealed container of processes at equilibrium. In fact, an articulated signaling network affecting the level of the involved enzymes occurs, making this pathway a relevant metabolic checkpoint for the cell health, in which different intermediates are functional links between the formation of NADPH and R5P and the different metabolic needs (Figure 2). Both TKT and TAL, which are differently represented in different tissues [64], catalyze relevant regulatory steps able to fulfil the main goals of PPP (i.e., to guarantee reducing power for anabolic demand and antioxidation protection and to allow nucleotides synthesis). The interplay of the enzymes involved in the two branches of PPP appears peculiar for different cells systems, leading, when altered, to cell type-specific pathologies [62,65]. Indeed, both the deficiency and the overexpression of TKT and TAL appeared to be linked to pathological states characterized by cell proliferation and cell death.

Transketolase-like 1, one of the mutated transketolase transcript isoforms of TKT, was found overexpressed in a variety of tumors. Gastric, uterine, colorectal, endometrial, renal, thyroid and liver cancer [66,67,68,69,70,71,72] are characterized by an elevated level of the enzyme, which was suggested as a marker of poor prognosis of the tumor and as potential antitumor target. TAL and G6PDH, are over expressed in different hepatomas [62]. The overexpression of these enzymes, which catalyze limiting steps of the two branches of the PPP, should fulfil the increased need of nucleotide synthesis for tumor cells. This would be accomplished through the channeling of glucose towards R5P synthesis from both the oxidative and the reversible arms of the pathway. The former would proceed through NADP^+^ reduction and the latter would favour, through the TKT-dependent generation of Er4P, the utilization of the glycolytic intermediates F6P and Ga3P [64]. Indeed, the interconnection between G6PDH and TAL appears to occur both on a structural and a functional basis, being the two enzymes in human neutrophils involved in a supramolecular structure, which appears to control the overall flux of the PPP [73]. This event led to hypothesize that the two enzymes are part of a functional strategic device devoted to equilibrate the two arms of the PPP. In such a competitive game, however, as predicted by a theoretical analysis of factors affecting the fluxes of G6P metabolism in red blood cells [74], TAL overexpression appears to act as a dominating factor in determining an overall decrease in NADPH production and thus a loss of antioxidant ability [64]. On the other hand, in Jurkat human leukemic T cells, the overexpression of TAL is accompanied by a deficiency of both G6PDH and 6PGDH. In this case, no competition between the two PPP branches occurs and, as predictable, a concomitant significant loss of NADPH, reduced levels of GSH and high rate of ROS formation were observed. These cells are prone to apoptotic events induced by H_2_O_2_ and NO, by signaling factors as tumor necrosis factor-α, anti-FAS monoclonal antibodies or simply by serum deprivation [75].

As it occurs for overexpression of TAL, the activation of the enzyme through phosphorylation resulted in a depletion of NADPH and induction of oxidative stress in fibroblasts from patients affected by Xeroderma pigmentosum or transformed by SV40. In these cell systems, the apparent involvement of TAL in the homeostasis of cell reduced state is concomitant with a low activity of catalase, another antioxidant enzyme whose activity could be restored by NADPH supplementation [76].

Not only hyperactivity, but also a reduced activity of TKT and TAL has a significant impact on cell function. A reduced activity of TKT was shown in neurodegenerative diseases, diabetes mellitus and cancer [77,78] and associated in diabetes to an increase in circulating ribose and glycation phenomena. Indeed, while the complete deficiency of TK and G6PDH is lethal, TAL deficiency may be tolerated by some cell types and tissues, even though the human organism cannot survive in the absence of this enzyme. TAL deficiency is associated to a variety of different pathologies, as multiple sclerosis and rheumatoid arthritis as well as different tumors [79]. It is remarkable how the modulation of NADPH and ROS levels, exerted by TAL activity, affects the mitochondrial trans-membrane potential [79]. In this regard, both TKT and, more incisively, TAL deficiency were reported to activate in vivo the mitochondrial unfolded protein response (UPRmt) in Caenorhabditis elegans. In particular, it was shown how TAL deficiency determines, through complex oxidative stress and a starvation-like responses, mitochondrial morphology changes, impairment of mitochondrial respiration and decreased fats level, and increases the longevity of the animal. To conclude, the impact of TAL activity in modulating NADPH levels and controlling the ROS production mainly associated to mitochondrial oxidative processes, appears the result of the ability of the enzyme to favour R5P accumulation. In the case of TAL overexpression, R5P accumulation would occur through the recruitment of glycolytic precursors. In the case of TAL deficiency, the impairment of the recycle of R5P into G6P, limits the efficiency of the oxidative branch of PPI. In this respect, the accumulation of neurotoxic molecules, as erythritol, arabitol and ribitol, concomitant with a marked decline of NADPH levels, led to propose the involvement in the process of aldose reductase [80], a NADPH-dependent enzyme for which four and five carbon atom aldoses, including ribose, are indeed better substrates than glucose [81].

We can complete the survey on the reversible branch of PPP by looking at the remaining two enzymes, namely Ru5PI and Ru5PE.These enzymes, through their action on Ru5P, connect the two branches of the pathway. In fact, the two reaction products, Xu5P and R5P, can be addressed towards G6P resynthesis, thus allowing the complete utilization of the glucose molecule and then the maximization of NADPH generation. On the other hand, R5P is the indubitable precursor for nucleotide synthesis either de novo, through further activation of R5P to PRPP, or salvage, through its isomerization to R1P by phosphopentomutase [82,83]. As mentioned above, also in this case, the flux of intermediates of the reversible branch of PPP is not functionally isolated. In fact, the epimerization of Ru5P to Xu5P affects lipogenesis, being Xu5P (together with glucose and fructose-1,6-bisphosphate) the inducer of lipogenic genes through the activation of a protein serine/threonine phosphatase acting on the ChREBP (Carbohydrate-responsive element binding protein) transcription factor [84,85,86]. Also, the efficiency of the isomerization reaction between Ru5P and R5P may enter in this metabolic control, which may dangerously evolve to cancer when significantly stressed, as reported, for instance, for Ru5P-isomerase overexpression in human hepatocellular carcinoma [87,88]. In this regard, we may conclude underlining that a body of experimental evidence merge in the conclusion that dysregulation of the PPP promotes carcinogenesis [89].

All of these considerations evidence that an intervention at any point on PPP, which alters the level of an intermediate, may affect a complex body of processes whose relevance in being damaging or even beneficial on health has not yet been fully clarified [90]. Thus, ribose intake, forcing the generation of R5P by a high substrate concentration, besides pushing toward nucleotide synthesis, would influence PPP with not yet clearly assessed consequences. If this practice may be in principle understandable or even acceptable in severe pathological situations as described above, in which “possible” damage is overcome by “ascertained” damage linked to the pathological status, it is not anymore understandable when it is applied to healthy, even “tired” subjects. On the basis of the above considerations, it should be clear when and why the ribose supply might be beneficial. “When”, in the case the generation of pentose phosphates through the G6PDH/6PGDH couple is knockdown by a failure of the oxidative PPP and/or in the case of a failure of the reversible PPP branch in recruiting glycolysis intermediate for R5P synthesis. “Why”, in the attempt to counteract an emergency. It should be obvious, however, that the treatment should occur under a strict medical control and for a proper limited time. This is due to the fact that the metabolic and functional “price” of such a therapeutic approach may be high (see below).

## 5. The Shady Side of the Ribose Intake

A relevant and deleterious feature of ribose is its glycative potential. When the question of protein glycation cannot be avoided, “ribose lovers” minimize the ribose features presenting this molecule as a natural sugar which, as it occurs for glucose, may induce protein glycation phenomena. This is true, but what is usually missed in the presentation of the molecule is that ribose is one of the sugars most effective in determining glycation reactions [91,92,93,94,95,96]. Indeed, protein glycation protocols are devised which make use of ribose to enhance glycation phenomena. Just as an example, in order to evaluate the influence of glycosylation on the progression of spontaneous osteoarthritis, the elective method to increase cartilage glycation was a topic injection of ribose in the knee of the animals [97]. Moreover, pentosidine, initially suggested as an index of protein glycation linked to dysmetabolism of pentoses [98] and nowadays considered one of the general biomarkers of advanced glycation end products (AGEs) [99], was firstly discovered as a product of the non-enzymatic reaction between ribose and lysine or arginine, and then formed in vitro by the reaction of ribose and other pentoses with human collagen [100].

**Figure 3:**
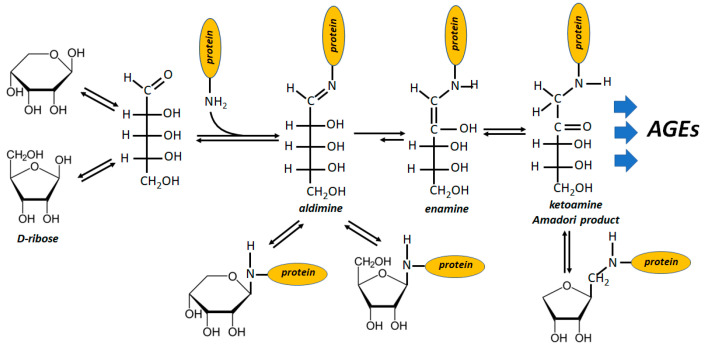
Schematic representation of AGEs generation upon protein glycation induced by D-ribose. The same reactions progression may apply to R5P, except for the pyranosidic structure of the sugar phosphate, for the cyclic N-substituted-1-C- ribopyranoside and then for the cyclic N-substituted-1-C-ribulofuranoside.

Protein glycation starts from the reaction of the free aldehydic group of sugars with protein amino residues and the special glycation ability of ribose stands on the high level of free aldehydic form which accounts for 20 to 30 times that of glucose [101] (Figure 3). In fact, R5P is unable to form a pyranosidic structure and displays an even higher availability of free aldehydic groups with respect to ribose [102,103]. It is not surprising, therefore, that also R5P is an efficient inducer of the Maillard reaction sequence (even more active than ribose, being 100-fold faster than glucose) [104]. Moreover, R5P enters the glycation mechanism with its phosphate group, which absolves a catalytic function in the products formation, promoting, at the same time, oxidative stress [103,105,106].

Many proteins have been found to be extremely susceptible to glycation by ribose, a process which evolves with generation of deleterious AGEs [91]. The glycating action on hemoglobin [92,107] and myoglobin [108,109] is remarkable. The deleterious effects induced by ribose glycation on other functional proteins are also widely documented. Thus, ribose-induced glycated serum was found to be toxic towards cultured pancreatic β-cells in terms of viability and insulin secretion [110]. Ribose, besides targeting albumin [93,111,112], was shown to actively glycate other plasma proteins, such as fibrinogen [113], low density lipoprotein [114,115,116], and immunoglobulin-G [96]. Also, neuro degenerative disorders find in AGEs generation a potent eliciting factor [117,118]. Thus, neuronal Tau-protein, α-synuclein, β2-microglobulin, following glycation, contribute to the development of several common neurodegenerative diseases [119,120,121,122,123,124]. In addition, cognitive impairments observed in animal models and in diabetic patients has been ascribed to glycation and AGEs accumulation linked to ribose [93,125,126,127,128,129,130]. Diabetic nephropathy also appears linked to glycation processes and AGEs accumulation [131,132,133]. Collagen is another possible glycation target with consequences in the proper function of bones and tendons [134,135,136,137]. It is rather difficult to find tissues or pathological situations in which the deleterious effect of not enzymatic glycation and AGEs accumulation can be excluded. Also, DNA and DNA related enzymes have been found to be glycated by ribose, with consequences that, speaking generally, are difficult to predict [116,138,139,140,141,142].

The source of the above underlined phenomena should be ascribed to the level of circulating ribose. The blood level of free ribose is rather low, resulting in fasting condition 50 times lower than glucose. Whether this may be related to the efficient preservation of the hemiacetal furanosidic scaffold of the molecule (i.e., R1P, as it occurs in the nucleotide salvage pathways), or to its high reactivity or both, is a debatable and open question. What appears indubitable, however, is the evident plan of nature to avoid this cytotoxic molecule standing around in the organism and, at the same time, fulfilling the need of the ribose scaffold making use of glucose (a more reliable and safer molecule) [1,8].

As for glucose, the ribose levels in blood and urine increase in diabetes, a fact shown to correlate with haemoglobin glycation and with a deficiency of TKT [107,143]. This evidence strongly supports the view of ribose as a relevant factor in the development of diabetes and related complications [95,107,144].

It is worth noting that the above list of references is only part of the vast field of reports dealing with the effects associated with glycation phenomena and AGEs accumulation induced by ribose.

**Figure 4:**
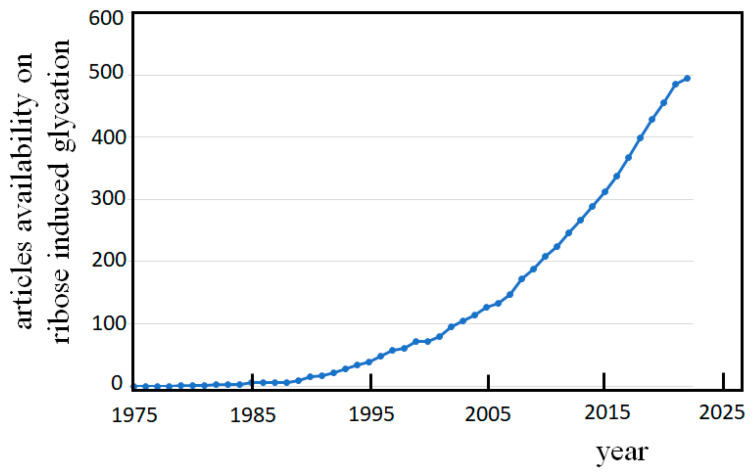
Articles availability along the past forty years. Numbers emerge from the PubMed^®^ data base, searching for: “glycation and ribose” on August 2022.

The number of publications on the damaging inference of these events during the past forty years, as emerges simply searching for the topic “glycation and ribose” in PubMed^®^, (Figure 4) clearly shows that the consciousness is arising within the scientific community regarding the risky phenomenon of ribose glycation.

Thus, it is somehow disturbing that even nowadays there are papers dealing with the effects of ribose administration in human in which the words “glycation” or “AGEs” are lacking, especially when the conclusion is suggestive of the good and safe practice for healthy people to assume ribose to feel better. On the other hand, a similar lack of consciousness is evident when the risky phenomenon of ribose-glycation is experimentally faced. Emblematic in this respect are the results coming from administration of ribose to horses [4]. This paper, which is frequently cited as a reference in commercial advertisement inserts supporting ribose as a dietary integrator, reports that the treated animals felt better and no glycation phenomena were observed. In conclusion, ribose intake is satisfactory for the horses and should be good also for human.

Considering the glycation ability of ribose, we do believe that such an unexpected result is, at least, too hazardous in suggesting a safe use of ribose in humans.

Unfortunately, the claim for a safe and desirable use of ribose as food integrator is not so infrequent as conclusion statement in the literature dealing with ribose intake. So, the feasibly harmful action of ribose remains underestimated in the commercial world.

## 6. Conclusions

Ribose is a cytotoxic molecule whose availability in the cell is apparently kept under rigorous control. Clear evidence shows the benefits of intervention of ribose in peculiar distress situations as it may occur in ischemia, cardiomyopathy, and hypertrophy. However, if one is to consider ribose as a potential therapeutic tool in conditions in which the molecule may relieve the patient from discomfort, one must think to administer ribose as an integrator to healthy people. In the former case, we face, under a rigorous medical control, a damaging distress situation in which both patient and physician are aware of the balance between benefits and side effects. On the contrary, in the case of the use of ribose as food integrator, based on the suggestion to become more energetic and stronger, the users are driven only by the unlimited desire of humans to feel better. Ribose is therefore a quite interesting and intriguing molecule, but much more experimental effort is needed to safely enjoy of it, if even it will be the case.

## Data Availability

Not applicable.
